# Eye-Size Variability in Deep-Sea Lanternfishes (Myctophidae): An Ecological and Phylogenetic Study

**DOI:** 10.1371/journal.pone.0058519

**Published:** 2013-03-05

**Authors:** Fanny de Busserolles, John L. Fitzpatrick, John R. Paxton, N. Justin Marshall, Shaun P. Collin

**Affiliations:** 1 Neuroecology Group, School of Animal Biology and the Oceans Institute, The University of Western Australia, Crawley, Western Australia, Australia; 2 Centre for Evolutionary Biology, School of Animal Biology, The University of Western Australia, Crawley, Western Australia, Australia; 3 Computational and Evolutionary Biology, Faculty of Life Sciences, University of Manchester, Manchester, United Kingdom; 4 Ichthyology, Australian Museum, Sydney, New South Wales, Australia; 5 Sensory Neurobiology Group, Queensland Brain Institute, University of Queensland, Brisbane, Queensland, Australia; Lund University, Sweden

## Abstract

One of the most common visual adaptations seen in the mesopelagic zone (200–1000 m), where the amount of light diminishes exponentially with depth and where bioluminescent organisms predominate, is the enlargement of the eye and pupil area. However, it remains unclear how eye size is influenced by depth, other environmental conditions and phylogeny. In this study, we determine the factors influencing variability in eye size and assess whether this variability is explained by ecological differences in habitat and lifestyle within a family of mesopelagic fishes characterized by broad intra- and interspecific variance in depth range and luminous patterns. We focus our study on the lanternfish family (Myctophidae) and hypothesise that lanternfishes with a deeper distribution and/or a reduction of bioluminescent emissions have smaller eyes and that ecological factors rather than phylogenetic relationships will drive the evolution of the visual system. Eye diameter and standard length were measured in 237 individuals from 61 species of lanternfishes representing all the recognised tribes within the family in addition to compiling an ecological dataset including depth distribution during night and day and the location and sexual dimorphism of luminous organs. Hypotheses were tested by investigating the relationship between the relative size of the eye (corrected for body size) and variations in depth and/or patterns of luminous-organs using phylogenetic comparative analyses. Results show a great variability in relative eye size within the Myctophidae at all taxonomic levels (from subfamily to genus), suggesting that this character may have evolved several times. However, variability in eye size within the family could not be explained by any of our ecological variables (bioluminescence and depth patterns), and appears to be driven solely by phylogenetic relationships.

## Introduction

The detection of light signals is one of the most important means by which organisms perceive and react to their surroundings. In bony fishes (infraclass Teleostei), like all other vertebrates, light detection is achieved by a highly conserved structure, the eye, in which a single lens focuses an image on to the neural retina. Since the cornea is not refractive underwater [1], the lens of most teleosts is spherical and its radius and the distance from the lens centre to the retina (focal length) are related by a “constant” defined, over 100 years ago, as Matthiessen's ratio [2], [3], regardless of the size of the eye. Thus, the acuity (quality of the image) of the teleost eye is directly related to eye size and lens diameter but is also influenced by other factors such as the quality of the optics, the amount of light received (governed by the aperture or pupil size) and the degree of overlap between the dendritic fields of neighbouring retinal receptors. As visual acuity is limited by the amount of light available, the evolution of the visual system in teleosts is also expected to be influenced by the depth at which an individual lives.

The mesopelagic zone (200–1000 m), also referred to as the twilight zone, is characterised by exponentially diminishing levels of downwelling sunlight. Although the low amount of sunlight in this zone negates the process of photosynthesis, enough light is present in the water column to create an extended visual scene both vertically and horizontally [4], thereby allowing animals to detect the silhouettes of potential prey items against a lighter background when viewed from below [5]. The mesopelagic zone also contains the greatest biomass and diversity of animals in the mid-waters of the ocean [6], many of which are bioluminescent, producing light signals using either symbiotic bioluminescent bacteria (i.e. anglerfish [7]) or their own enzymatic complex (luciferin-luciferase, i.e. viperfish [8]). Due to the low levels of sunlight and the predominance of small bioluminescent flashes, vision is considered very important in the twilight zone and seems to be the dominant sense used by its inhabitants [9]. To be able to see in dim conditions and for viewing bioluminescence, the visual system of mesopelagic fishes requires higher sensitivity than acuity [10]. To enhance sensitivity, species have adapted to optimize light collection and extend the visual field [11]. One of the most extreme ocular adaptations at these depths (200–1000 m) is the tubular shape of the eyes of a number of species (i.e. *Argyropelecus* sp., *Winteria* sp.), which are directed upwards (and rostrally in some species) to optimise light capture of the downwelling sunlight [12], [6], [13], [14]. However, the most important morphological adaptation of the visual system of mesopelagic fishes is arguably the enlargement of their eyes compared to body size [15]; a larger eye will increase the chance of photon capture (greater pupillary aperture), thereby allowing improved detection of body silhouettes and bioluminescent flashes against an increasingly dim background [5], [1], [16].

Deeper in the ocean, in the bathypelagic zone (1000–4000 m), where no downwelling sunlight penetrates, eye size tends to decrease with some species of teleosts having such small eyes they were considered “degenerate” [17]. The reduction in eye size in inhabitants of the bathypelagic zone could be explained by the predominant use of other sensory systems and/or the brighter appearance of the bioluminescent signals in the absence of residual downwelling sunlight. The bathypelagic zone represents a visual scene composed only of bright, point sources of light viewed against a completely dark background. To detect these intermittent sources of light, the eyes do not need to be very large as the flashes will appear extremely bright compared to the background [16]. In addition to the absence of residual daylight, the bathypelagic zone is also characterised by a drastic diminution in animal biomass and biodiversity [6]. An individual living in this zone will encounter fewer animals and therefore less bioluminescent signals (the only type of light present in the bathypelagic zone). In this zone, vision may be used secondarily after a potential prey or mate has been detected using other sensory modalities.

More than half a century ago, Marshall [15] compared the eye size of three species of *Gonostoma* living in different oceanic zones, the upper mesopelagic (*G. denudatum*), lower mesopelagic (*G. elongatum*) and the bathypelagic (*G. bathyphilum*) zones and observed that the deeper the species the smaller the size of the eye. For species that are restricted to a particular zone, this inverse relationship between depth and eye size appears intuitive. However, some species may frequent all three zones (epipelagic, mesopelagic, bathypelagic) during their lifetime or even within the same day (diel vertical migrations). How does eye size vary in these species? Despite the importance of considering depth, in terms of light availability, when evaluating the evolution of the visual system in deep-sea fishes, it remains unclear how eye size is shaped by depth and other environmental conditions. Consequently, in this study we aim to assess to what extent variability in eye size can be explained by ecological differences in habitat and lifestyle within a single family of fishes characterized by broad intra- and interspecific variance in depth range and luminous patterns. Specifically, we focus our investigation on lanternfishes (family Myctophidae), one of the most abundant group of mesopelagic fishes in the world's oceans [18], with some 250 species in 33 genera currently recognised [19]. Most species exhibit extensive diel vertical migration toward the surface at night in order to feed and a great interspecific variability in their migration patterns has been observed [20]. Individuals can migrate from the epipelagic zone at night to the upper part of the bathypelagic zone during the day. Myctophids produce bioluminescence using a luciferin-luciferase reaction [21], [22]. They possess two kinds of luminous structures, with highly variable patterns, that light up independently. Those are the ventral and ventrolateral photophores which are thought to play a role in counter-illumination [23] and species recognition [24], and the luminous organs and tissue patches, which are frequently sexually dimorphic, are located on the caudal peduncle and/or head and/or body and may play several different roles including intra- and interspecific communication, prey illumination and distraction [25]. Consequently, we hypothesised that lanternfishes with a deeper distribution and/or a reduction of bioluminescent emissions will have smaller eyes and that ecological factors rather than phylogenetic relationships will drive the evolution of their visual system. We test these predictions by investigating the relationship between the relative size of the eye (corrected for body size) and variations in depth and/or luminous-organ patterns in the Myctophidae using phylogenetic comparative analyses.

## Materials and Methods

### Ethics statement

For cruises 1–4 (Table 1), sampling was carried out under the following collection permits: Coral Sea waters (CSCZ-SR-20091001-01), Commonwealth waters (AU-COM2009051), GBRMPA (G09/32237.1) and Queensland Fisheries (133805), (Marshall, AEC # SNG/080/09/ARC). For cruises 5–6, sampling permits were obtained by the Chief Scientist of the respective cruises (AIMS, University of Tübingen) for their target species. We obtained our samples as by-catch and therefore no collection permits were required. Most individuals caught were already deceased; however, moribund animals were humanely euthanized following the guidelines of the NH&MRC Australian Code of Practice, under our University of Western Australia Animal Ethic protocol (RA/3/100/917). Tissue samples obtained from collaborators (cruises 7–9) did not require any UWA collection or animal ethics permits.

**Table 1:** Summary of the research cruises from which the samples were collected, together with their geographic location, fishing equipment, time of sampling and fixative used.

Cruise	Location	Date	Equipment	Time of sampling	Fixation
1	Coral Sea	11/2009	RMT*/plankton net/neuston net	Night	4% PFA, Karnovsky
2	Off Osprey reef, Coral Sea	05/2010	IKMT	Night	4% PFA, Karnovsky
3	Coral Sea	12/2010	RMT*	Night	4% PFA, Karnovsky
4	Off Osprey reef, Coral Sea	06/2011	IKMT	Night	4% PFA, Karnovsky
5	Off Osprey reef, Coral Sea	07/2012	IKMT	Night	4% PFA, Karnovsky
6	Peru-Chile Trench	09/2010	RMT*/neuston net	Day, Night	5% formalin, 2% glutaraldehyde
7	Bay of Biscay, North East Atlantic	10/2009	Bottom trawl GV100^1^	Day	Karnovsky
8	Balearic Islands, Western Mediterranean Sea	07/2010	Double-warp modified commercial mid-water trawl/IKMT^2^	Day, Night	Karnovsky
9	Western Australia	07/2010	EZ net*	Night	5% formalin

1 Sourced from Olivar et al. [74],

2 sourced from Mahé & Poulard [75]. EZ = multiple plankton net system; IKMT = Isaacs-Kidd Midwater Trawl; RMT = Rectangular Midwater Trawl.

* Indicates the use of an opening-closing device.

### Data collection and morphometric measurements

A total of 237 lanternfishes from 61 species and 19 genera were analysed in this study. Samples were obtained from different research cruises in different parts of the world and with different methods of sampling (Table 1). For each individual, the standard length, rostro-caudal eye diameter (measured *in situ*) and lens diameter were measured with digital calipers to 0.1 mm. For most of the lanternfish, measurements were performed on fresh specimens on board ship prior to fixation. However, when samples were acquired from collaborators (Cruises 7, 8, 9, see Table 1), the measurements were made after fixation. The fixatives used in those cases were 5% buffered formalin and Karnovsky's fixative (2.5% paraformaldehyde and 2% glutaraldehyde in 0.1 M cacodylate buffer). Since we were interested in the eye size: body size relationship and because formaldehyde has previously been found to affect body length by only 0.8% [26], no measurement correction was used between fresh and fixed specimens and all the morphometric data for each species were pooled. The life stage of each specimen was estimated by length measurements published in the literature. With the exception of sexually dimorphic features, juvenile and adult lanternfishes are identical in appearance (i.e. their body proportions, pigment and photophore patterns, [27]) and since the regression slopes of eye diameter versus standard length were not significantly different between the two stages (Table S1), these two groups were analysed together. One specimen of *Scopelengys tristis* of the family Neoscopelidae, sister family of the Myctophidae, was also measured for comparison.

### Taxonomic remarks

Most of the samples from the Coral Sea (Cruises 1–5) and Chile-Peru Trench (Cruise 6) are registered as voucher specimens at the Australian Museum in Sydney, Australia. However, further taxonomic analyses need to be carried out for six of our study species to confirm identification. A comment for each of these six species is given below. *Lampanyctus vadulus*: requires confirmation of some northeastern Australian variants of *L. nobilis* currently under study. *Myctophum spinosum*/*M. lychnobium*: the characters distinguishing these two species appear to form a continuum in the western South Pacific; the specimens studied here are the two extremes of the continuum that might be all *M. spinosum*. *Symbolophorus cf. boops*: *Symbolophorus* from the eastern South Pacific require more study to allow species identification. *Nannobrachium cf. nigrum*: the specimens used match the description in Zahuranec [28], but not the figure and brief description of the holotype in Nafpaktitis et al. [29]. *Triphoturus oculeus*: detailed examination of the specimens from the eastern South Pacific to distinguish any possible *T. mexicanus* has not been completed to date.

### Ecological data

A dataset of the location and sexual dimorphism of the luminous tissue was created using information found in the literature (Table 2). Presence-absence of luminous organs (head, caudal), additional luminous patches and sexual dimorphism in luminous tissues were noted [30], [31], [29], [32], [28], [33]. All lanternfish genera possess a dorsal nasal organ (Dn) and/or a ventral nasal organ (Vn) on their head associated with the eye. Only species presenting an enlarged Dn and/or Vn were given a special category in this study. Depending on the species, sexual dimorphism in luminous tissues can be seen in the Dn/Vn, caudal luminous organs and/or luminous patches (Table 2). Differentiation of the type of sexual dimorphism in our analyses did not show significant differences. As a consequence, only results for the presence/absence of sexually dimorphic features are presented in this study (Table 2). For each species, the juvenile/adult depth distributions during night and day were recorded, taking into consideration the individual size and area of sampling when possible (Table S2, S3). Published studies using opening-closing sampling devices [34], [27], [35], [36], [37], [38], [39] and differentiating life stages [40], [27], [35], [38] were given priority when making the dataset. Categorised depth ranges were created for the statistical analyses based on the published data and our own capture-depth data. The same number of depth classes was used for both diurnal and nocturnal depth distribution in order to statistically compare the results. The group's cut off, during day and night, was chosen at the most meaningful depth in terms of vision and accuracy of the depth data. Three group categories were created in terms of the amount of downwelling light present: moderate light level (0–5 m at night, 200–500 m during the day), low light level (5–100 m at night, 500–900 m during the day), no light (<100 m at night, <900 m during the day). For the night-depth range, species (excluding larvae) were classified according to their shallowest depth recorded. In the surface category at night, only species dip-netted or sampled with a neuston net (surface) were included. For the day-depth range, species (excluding larvae) were classified according to their deepest depth recorded.

**Table 2:** Dataset used in the phylogenetic comparative analyses of the location of the luminous tissue (Dn/Vn, Caudal, Body patches), whether there was any level of sexual dimorphism present (Sex. dim. Sex. D., Sex. C. Sex. P.) and the depth categories during night and day.

Species	Dn/Vn	Caudal	Patches	Sex. dim.	Sex. D.	Sex. C.	Sex. P.	Night	Day
*Benthosema glaciale*	0	1	0	1	0	1	0	1	1
*B. suborbitale*	0	1	0	1	0	1	0	1	1
*Bolinichthys longipes*	0	1	1	1	0	0	1	1	1
*B. nikolayi*	0	1	1	0	0	0	0	2	1
*B. supralateralis*	0	1	1	0	0	0	0	1	1
*Centrobranchus andreae*	0	1	0	1	0	1	0	2	1
*Ceratoscopelus maderensis*	0	1	1	0	0	0	0	1	2
*C. warmingii*	0	1	1	0	0	0	0	1	2
*Diaphus brachycephalus*	1	0	0	1	1	0	0	1	1
*D. danae*	1	0	1	0	0	0	0	1	1
*D. fulgens*	1	0	1	1	1	0	1	1	2
*D. garmani*	1	0	1	1	1	0	0	1	1
*D. holti*	1	0	1	1	1	0	0	1	1
*D. luetkeni*	1	0	1	1	1	0	0	1	1
*D. meadi*	1	0	1	1	1	0	1	1	2
*D. mollis*	1	0	1	1	1	0	0	1	1
*D. parri*	1	0	1	1	1	0	0	1	1
*D. phillipsi*	1	0	1	0	0	0	0	1	1
*D. regani*	1	0	1	0	0	0	0	1	2
*D. splendidus*	1	0	1	1	1	0	0	1	1
*D. termophilus*	1	0	1	1	1	0	0	1	1
*D. whitleyi*	1	0	1	1	1	0	0	2	1
*Diogenichthys atlanticus*	1	1	0	1	1	1	0	1	2
*D. laternatus*	1	1	0	1	1	1	0	0	0
*Electrona risso*	0	1	0	1	0	1	0	1	1
*Gonichthys tenuiculus*	0	1	0	1	0	1	0	0	n.a.
*Hygophum benoiti*	0	1	0	1	0	1	0	1	2
*H. hygomii*	0	1	0	1	0	1	0	1	2
*H. proximum*	0	1	0	1	0	1	0	0	1
*Lampadena luminosa*	0	1	0	0	0	0	0	1	2
*L. urophaos*	0	1	0	0	0	0	0	1	1
*Lampanyctus alatus*	0	1	1	0	0	0	0	1	1
*L. crocodilus*	0	1	1	0	0	0	0	1	2
*L. iselinoides*	0	1	1	0	0	0	0	1	1
*L. nobilis*	0	1	0	0	0	0	0	1	2
*L. omostigma*	0	1	0	0	0	0	0	1	0
*L. parvicauda*	0	1	0	0	0	0	0	1	2
*L. pusillus*	0	1	0	0	0	0	0	1	2
*L. vadulus*	0	1	0	0	0	0	0	1	2
*Lobianchia dolfleini*	0	1	0	1	0	1	0	1	1
*L. gemellari*	0	1	0	1	0	1	0	1	1
*Loweina interrupta*	0	1	0	1	0	1	0	1	n.a.
*Myctophum asperum*	0	1	0	1	0	1	0	0	1
*M. aurolaternatum*	0	1	0	1	0	1	0	0	2
*M. brachygnathum*	0	1	0	1	0	1	0	0	0
*M. lychnobium*	0	1	0	1	0	1	0	0	0
*M. nitidulum*	0	1	0	1	0	1	0	0	1
*M. obtusirostre*	0	1	0	1	0	1	0	0	1
*M. spinosum*	0	1	0	1	0	1	0	0	1
*Nannobrachium cf. nigrum*	0	1	0	0	0	0	0	2	1
*N. idostigma*	0	1	0	0	0	0	0	1	0
*N. phyllisae*	0	1	0	0	0	0	0	2	n.a.
*Notolychnus valdiviae*	0	1	0	1	0	1	0	1	2
*Notoscopelus elongatus*	1	1	1	1	0	1	0	1	2
*N. kroeyerii*	1	1	1	1	0	1	0	1	2
*Symbolophorus cf. boops*	0	1	0	1	0	1	0	0	1
*S. evermanni*	0	1	0	1	0	1	0	0	1
*S. rufinus*	0	1	0	1	0	1	0	0	1
*S. veranyi*	0	1	0	1	0	1	0	0	1
*Triphoturus nigrescens*	0	1	0	0	0	0	0	2	2
*T. oculeus*	0	1	0	0	0	0	0	1	1

Sex. dim. = sexual dimorphism in luminous tissue, Sex. D. = sexual dimorphism in Dn/Vn luminous organs, Sex. C. = sexual dimorphism in caudal luminous organs, Sex. P. = sexual dimorphism in luminous tissue patches, 0 = character absent, 1 = character present. For the night depth range, 0 = 0–5 m, 1 = 5–100 m, 2 = <100 m. For the day depth range, 0 = 200–500 m, 1 = 500–900 m, 2 = >900 m, n.a. = missing values.

### Phylogenetic analyses

**Figure 1:**
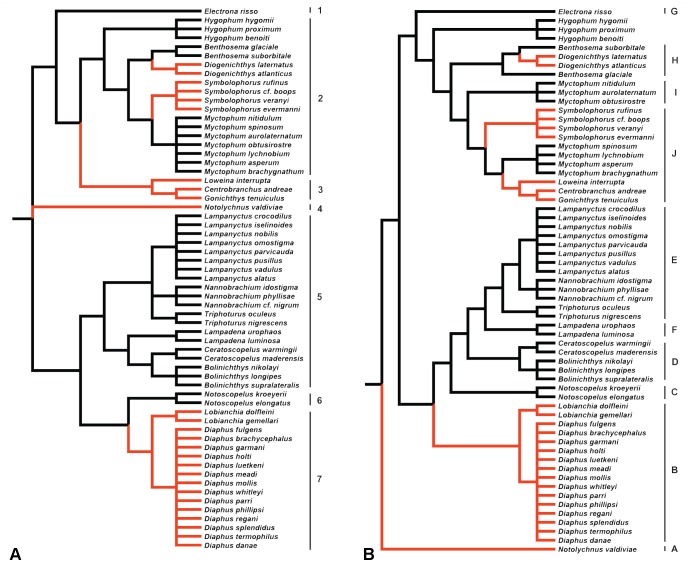
Phylogenies of Myctophidae reconstructed from (A) Paxton et al. [43], (B) Poulsen et al. [44]. The red branches indicate the main differences between the two phylogenies. Branch lengths are arbitrarily ultrametricized on the figure. In A, the numbers identify the different tribes of Paxton et al. [43], 1. Electronini, 2.Myctophini, 3.Gonichthyini, 4.Notolychnini, 5.Lampanyctini, 6.Gymnoscopelini, 7.Diaphini. In B, the letters identify the different clades of Poulsen et al. [44], A. Notolychnini, B. Diaphini, C. Notoscopelini, D-E-F. Lampanyctini, G. Electronini, H. Myctophini, I. Myctophini (cycloid-species-group), J. Myctophini (ctenoid-species group)+Gonichthyini.

Standard statistical analyses assume independence of the samples. This assumption is unfortunately not met when comparing different species as more closely related species are expected to be more similar to one another due to the share of a common ancestor. Therefore, all data analyses were performed using phylogenetic comparative analyses to account for the shared history among species [41]. Unfortunately, no fully resolved phylogeny is available for the family Myctophidae to date. Consequently, two different phylogenies, A and B (Figure 1), were built in the Mesquite program v. 2.75 [42] based on two published phylogenies [43], [44]. Paxton et al.'s phylogeny classified genera using derived character states of adult osteology and photophore patterns, as described by Paxton et al. [43], and of larvae as described by Moser and Ahlstrom [45], [46], [47]. The phylogeny divided the family into two subfamilies (Myctophinae and Lampanyctinae) and seven tribes (Electronini, Myctophini, Gonichthyini, Diaphini, Gymnoscopelini, Lampanyctini and Notolychnini, Figure 1A). The only difference between Paxton's originally described phylogeny and the one used in the present analysis (phylogeny A) is the inclusion of the genus *Nannobrachium*, which was added to Paxton's phylogeny after Zahuranec [28]. The phylogeny of Poulsen et al. [44] was the first molecular phylogeny for the family and used mitogenomic results from DNA sequences and unique gene orders from 38 lanternfish species. Poulsen et al. [44] confirmed the presence of the two subfamilies (Myctophinae and Lampanyctinae) and identified 10 monophyletic lineages or clades (Figure 1B). The genus *Hygophum* was added to Poulsen et al.'s originally described phylogeny in our study (phylogeny B), although no clade was assigned, and its position in the phylogeny was kept identical to Paxton et al.'s phylogeny. The main differences seen in Poulsen et al.'s phylogeny compared to Paxton et al's are the taxon *Notolychnus*, which became a sister taxon of all the remaining myctophids, and the tribe Diaphini, which became a sister tribe of the Lampanyctini. Due to the lack of resolution, both phylogenies are only resolved to generic level, resulting in several polytomies (i.e. unresolved relationship among species). Unfortunately, the presence of polytomies prevents the application of many phylogenetic analyses that require a fully resolved phylogeny. Therefore, to bypass this problem, 100 alternative phylogenies were generated with polytomies randomly resolved to infinitesimally small (10^−6^) branch lengths using the Mesquite program v. 2.75 [42]. Ten of these phylogenies with randomly resolved polytomies were selected at random to perform the different analyses and the results between each of the 10 phylogenies compared for consistency. Moreover, to fit the statistical requirements for the phylogenetic linear models described below, branch lengths were transformed using Grafen's method [48] with rho transformation set at 2.5 before all analyses. All statistical analyses were performed, using both phylogenies separately, on Log_10_-transformed species averages with the statistical program R v.2.15.0 (R Foundation for Statistical Computing 2012).

### Estimating phylogenetic signal

The phylogenetic signal for continuous and discrete traits was estimated with Pagel's lambda (λ) using the package GEIGER in R [49]. Pagel's λ is a measure of the degree of phylogenetic dependence in the data [50], meaning to which degree closely related species are more similar to each other than what is expected by random evolutionary processes. Pagel's λ varies from 0 to 1, with λ value of 1 indicating that traits gradually accumulate changes over time in a Brownian motion process (i.e. random change in any direction) and λ values of 0 indicating that no phylogenetic signal is present and that traits have evolved in response to selective processes. The observed λ value for each trait was compared to λ values of 0 and 1 using likelihood ratio tests with df = 1.

### Phylogenetic linear models

Relationships between morphological traits (eye diameter, lens diameter, body size) and the relationship between morphological and ecological traits were assessed using phylogenetic generalised least squares regressions (PGLS, [51]) with the package APE in R [52]. PGLS are classic generalised least squares regressions that additionally take into account the shared history of the different species by incorporating phylogenetic information into the analyses. PGLS regressions estimate a phylogenetic scaling parameter, λ, using maximum likelihood methods to determine the degree of covariance in the residuals of the model, while controlling for phylogenetic effects. This approach also examines if the scaling parameter λ significantly differs from 0 or 1 using likelihood ratio tests, where λ = 0 indicates no phylogenetic dependence in the data and λ = 1 indicates strong phylogenetic association in the data [50], [51]. PGLS models were used to assess the relationship between morphological traits, to identify if eye size differs between the two subfamilies (Myctophinae, Lampanyctinae) when correcting for the effects of body size, and to assess if eye diameter was related with various ecological parameters. Standard length was added as a covariate in all models.

### Phylogenetic ANOVAs

To identify differences at the tribal (phylogeny A), cladal (phylogeny B) and generic (phylogenies A and B) levels, phylogenetically corrected residuals of the eye diameter were calculated from eye diameter - standard length regression fit lines using PGLS. Statistical analyses on residuals are usually not recommended as they often lead to biased results, especially if the variables tested are colinear with the controlled variable [53]. However, when too many groups are present, phylogenetic ANCOVA (i.e. analysis of covariance incorporating phylogenetic information) cannot sort out the differences using the PGLS approach. Consequently, residuals were used in this particular case to estimate differences between groups (tribes, clades, genera) using phylogenetic ANOVA (i.e. a classic ANOVA incorporating phylogenetic information [54]) followed by a sequential Bonferroni post-hoc test using the GEIGER [49] and PHYTOOLS [55] packages in R. At the generic level comparison, both phylogenies A and B were used separately and the results compared. Only groups with at least three observations were included in those analyses. The genus *Hygophum* was excluded from the analyses at the cladal level due to its hypothesized position in Poulsen et al.'s phylogeny.

## Results

### Morphometric measurements

**Figure 2:**
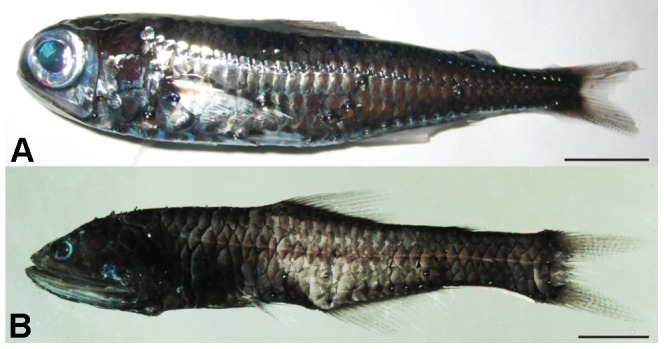
Difference in eye size compared to body size in two species of lanternfish. (A) *Myctophum brachygnathum*,(B) *Nannobrachium phyllisae*. Scale bar, 10 mm.

Eye size varied greatly within the lanternfish family (Figure 2). The range of values for standard length, eye diameter and lens diameter for each species is given in Table 3 in addition to the life stages and the origin of the specimens. In our dataset, standard length varies from 17.6 mm (*Diogenichthys laternatus*) to 126.4 mm (*Diaphus danae*); eye and lens diameter range from 1.5 mm to 11.1 mm and from 0.6 mm to 5.0 mm in *Nannobrachium idostigma* and *Myctophum lychnobium*, respectively.

**Table 3:** Range of standard length, eye diameter and lens diameter for each of the 61 species of Myctophidae analysed in this study.

Species	n	SL	Eye ø	Lens ø	stage	Cruise
*Benthosema glaciale*	1	42	4.6	1.8	A	8
*B. suborbitale*	2	28.2–29.0	3.0–3.1	1.3	A	9
*Bolinichthys longipes*	5	36.8–45.4	3.3–4.4	1.5–1.8	A	3
*B. nikolayi*	4	23.3–37.7	2.5–4.1	1.0–1.8	A+J	3
*B. supralateralis*	1	41.2	4.2	2.0	J	2
*Centrobranchus andreae*	1	31.5	2.2	0.8	?	1
*Ceratoscopelus maderensis*	1	52	5.1	2.1	A	8
*C. warmingii*	11	36.3–71.9	3.5–6.6	1.4–2.8	A+J	1, 3
*Diaphus brachycephalus* ^2^	2	33.9–35.2	4.1–4.3	1.6–1.8	A	3
*D. danae* ^1^	16	81.2–126.4	7.2–9.0	2.9–3.8	A	3
*D. fulgens* ^2^	1	41.2	3.9	1.8	?	2
*D. garmani* ^1^	3	32.8–42.1	2.4–3.5	1.0–1.5	A+J	4, 9
*D. holti* ^2^	1	40	5.1	2.1	A	8
*D. luetkeni* ^1^	5	35.2–40.8	2.3–2.9	2.4–3.1	A+J	1, 3
*D. meadi* ^2^	1	28.3	3.3	1.2	J	8
*D. mollis* ^2^	3	36.6–66.2	3.9–6.5	1.6–3.2	A	3, 9
*D. parri* ^2^	5	24.9–51.0	2.9–5.7	1.7–2.4	A+J	1, 2, 3, 4
*D. phillipsi* ^1^	4	26.2–29.5	2.1–2.4	1.0	J	3
*D. regani* ^1^	2	40.8–42.3	2.5–2.8	1.2–1.2	J	4
*D. splendidus* ^1^	4	26.7–48.1	1.5–2.8	0.6–1.3	A+J	3, 4
*D. termophilus* ^1^	6	48.3–77.3	4.2–6.5	1.9–3.2	A+J	1, 3, 4
*D. whitleyi* ^1^	2	59.5–90.3	4.0–5.5	1.7–2.6	A+?	3
*Diogenichthys atlanticus*	7	18.0–21.4	1.9–2.7	0.8–1.0	A	9
*D. laternatus*	11	17.6–31.1	2.0–3.2	0.7–1.3	A	6
*Electrona risso*	1	46	7.0	3.0	A	8
*Gonichthys tenuiculus*	3	40.6–49.4	2.7–3.5	1.3–1.6	A	6
*Hygophum benoiti*	1	45	6.2	2.3	A	8
*H. hygomii*	2	22.2–57.3	3.0–7.5	1.1–3.1	A+J	9
*H. proximum*	4	25.8–38.2	3.1–4.8	1.3–2.1	A+J	3, 4, 5, 6
*Lampadena luminosa*	1	104.3	8.4	3.7	A	3
*L. urophaos*	1	40	2.8	1.3	A	3
*Lampanyctus alatus*	9	30.3–49.8	1.7–3.3	?–1.3	A+J	3, 4, 9
*L. crocodilus*	1	31	1.7	0.6	J	8
*L. iselinoides*	1	34.3	2.0	0.8	J	6
*L. nobilis*	2	36.4–50.4	1.7–2.7	1.0	J	3
*L. omostigma*	1	27.8	1.9	0.7	?	6
*L. parvicauda*	9	28.4–106.0	1.7–6.4	0.9–2.8	A+J	6
*L. pusillus*	1	37	2.1	0.8	A	8
*L. vadulus*	4	37.4–81.8	2.2–5.2	0.9–2.12	A+J	1, 3, 4
*Lobianchia dolfleini*	2	27–33.3	1.9–2.2	1.0	A	8
*L. gemellari*	4	32.3–52.9	2.2–3.4	0.8–1.3	A+J	3
*Loweina interrupta*	1	25.9	2.0	0.9	J	9
*Myctophum asperum*	1	76.4	8.5	3.8	A	1
*M. aurolaternatum*	1	57.78	5.3	2.3	J	4
*M. brachygnathum*	7	65.7–69.7	6.8–7.6	1.7–3.6	A+J	2, 5
*M. lychnobium*	4	51.0–106.9	4.8–11.1	2.3–5.0	A+J	2, 4
*M. nitidulum*	11	25.5–85.4	2.3–7.1	0.9–3.2	A+J	6
*M. obtusirostre*	3	90.9–97.6	10.1–10.5	4.2–4.7	A	2, 4, 5
*M. spinosum*	5	32.7–87.5	3.2–8.7	1.4–3.9	A+J	1, 2, 4
*Nannobrachium cf. nigrum*	7	36.3–90.0	1.8–4	0.8–2.1	A	1, 3
*N. idostigma*	8	31.1–72.2	1.5–3.9	0.6–2.0	A+?	6
*N. phyllisae*	3	48.9–72.5	2.3–3.4	0.8–1.4	A+?	6
*Notolychnus valdiviae*	1	21.4	1.4	0.6	A	3
*Notoscopelus elongatus*	1	42	3.2	1.2	A	8
*N. kroeyerii*	4	95.2–105.1	6.1–6.7	2.7–2.9	A	7
*Symbolophorus cf. boops*	1	74.0	6.0	2.3	J	6
*S. evermanni*	13	33.6–65.8	2.5–6.2	1.0–2.8	J	2, 4
*S. rufinus*	8	30.5–73.7	2.1–6.2	1.2–2.9	J	2, 4
*S. veranyi*	1	85	6.7	2.9	A	8
*Triphoturus nigrescens*	1	35.4	2.0	0.9	A	3
*T. oculeus*	8	30–58.3	1.7–4.0	0.6–1.6	?	6

For each species, the sample size (n), the life stage (A = adult, J = juvenile) and the sample origin (Cruise) is given. For cruise number refer to Table 1. The superscript number for each *Diaphus* species indicates the group number made from the absence (1) or presence (2) of the So.

### Estimating phylogenetic signal

Estimation of the phylogenetic signal using Pagel's lambda gives relatively similar results with both phylogenies (Table 4). Results show that Dn/Vn and caudal luminous organs have a strong phylogenetic signal, suggesting that they gradually accumulate changes over time in a random evolutionary process. On the contrary, no phylogenetic signal is observed for the standard length and the day-depth distribution (phylogeny A only) variables. The other variables (eye diameter, lens diameter, residuals eye diameter, luminous patches, sexual dimorphism in luminous tissue and night depth distribution) show intermediate values of Pagel's lambda, which, although significantly different from 0 or 1, are generally closer to 1 depending on the phylogeny used. However, independent of the phylogeny used, the eye size corrected for body size (residuals eye diameter), the luminous patches and the luminous tissue sexual dimorphism variables show a strong phylogenetic signal very close to 1, again indicating that these traits changed randomly over time during lanternfish evolution (Table 4).

**Table 4:** Estimates of the phylogenetic signal for each variable using Pagel's Lambda.

Variables	λ (Phylogeny A)	λ (Phylogeny B)
Eye diameter	0.94^<0.001, <0.001^	0.75^0.02, <0.001^
Lens diameter	0.95^<0.001, <0.001^	0.79^0.02, <0.001^
Standard length	<0.001^1.0, <0.001^	<0.001^1.0, <0.001^
Residuals (eye/SL)	0.96^<0.001, <0.001^	0.94^<0.001, <0.001^
Dn/Vn organs	1^<0.001, 1.0^	1^<0.001,1.0^
Caudal luminous organs	1^<0.001, 1.0^	1^<0.001,1.0^
Luminous patches	0.99^<0.001, <0.001^	0.99^<0.001, <0.001^
Luminous tissue sexual dimorphism	0.93^<0.001, <0.001^	0.97^<0.001, <0.001^
Day depth	<0.001^1.0, 0.04^	0.79^0.113, <0.001^
Night depth	0.75^<0.001, <0.001^	0.67^<0.001, <0.001^

The results are presented for one of the ten randomly selected polytomy resolved phylogenies for the two different phylogenies. A λ value of 1 indicates that the trait gradually accumulates changes over time in a Brownian motion process. A λ values of 0 indicates that no phylogenetic signal is present and that traits have evolved in response to selective processes. The superscript values are likelihood ratio tests different from 0 and 1. Sample size is 61 for all variables except day depth (58).

### Relationship among morphometric traits

**Figure 3:**
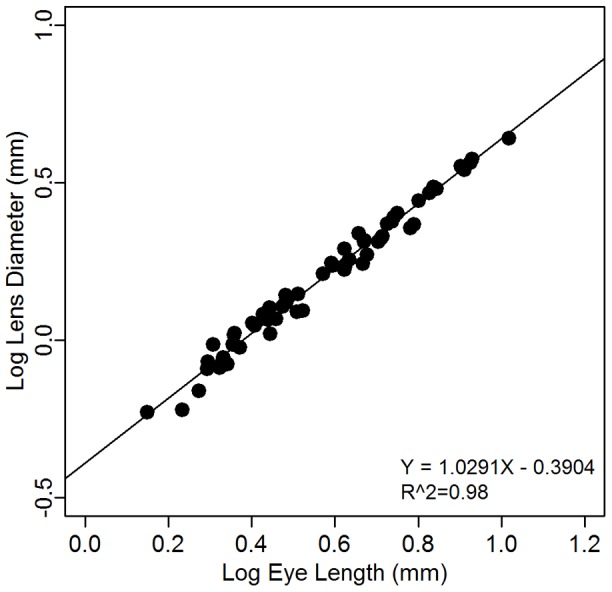
Relationship between lens diameter and eye diameter after correcting for phylogeny (PGLS). The fitted line is the linear regression corrected for phylogeny (PGLS) using the phylogeny of Paxton et al. [43].

**Figure 4:**
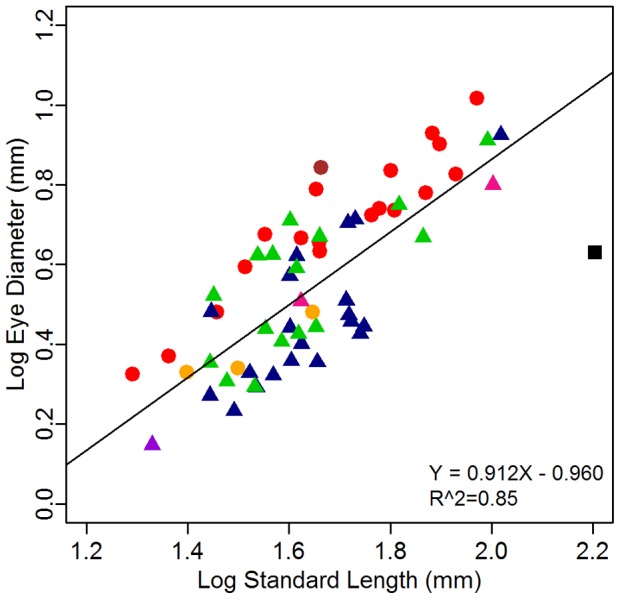
Relationship between eye diameter and standard length in 61 species of Myctophidae. Each point represents the mean for the species; individual details are in Table 2. Shapes represent the subfamilies, circles = Myctophinae, triangles = Lampanyctinae. Colors represent the tribes of Paxton et al. [43], brown = Electronini, red = Myctophini, blue = Lampanyctini, green = Diaphini, yellow = Gonichthyini, pink = Gymnoscopelini, purple = Notolychnini. The fitted line is the linear regression corrected for phylogeny (PGLS) using the phylogeny of Paxton et al. [43]. The black square represents one individual, *Scopelengys tristis*, from the sister family Neoscopelidae.

Phylogenetic linear regression shows that lens diameter is strongly, (positively) correlated with eye diameter (PGLS, n = 61, R^2^ = 0.98, *t-value* = 53.48, *P*≤0.001; Figure 3). Due to this close relationship between eye and lens diameter, we focused all subsequent analyses solely on the eye diameter – standard length relationship. Phylogenetic linear regression reveals that the eye diameter is positively correlated with standard length (PGLS, n = 61, R^2^ = 0.74, *t-value* = 13.05, *P*≤0.001, Figure 4). However, variations in eye size are observed between the different myctophid species, both at the level of subfamilies and tribes with representatives of the same subfamily or tribe having smaller or larger eyes (Figure 4). Probably due to this great variability, no significant difference was found between the two subfamilies (Myctophinae and Lampanyctinae) in terms of eye size (PGLS, n = 61, standard length effect: *t*_A_ = 12.95, *p*_A_≤0.001, *t*_B_ = 13.85, *p*_B_≤0.001; subfamily effect: *t*_A_ = 0.41, *p*_A_ = 0.68, *t*_B_ = 0.62, *p*_B_ = 0.54). The representative of the lanternfish sister family Neoscopelidae, *Scopelengis tristis*, showed a relatively small eye compared to all the Myctophidae analysed (Figure 4).

### Morphometric comparisons among tribes and clades

**Figure 5:**
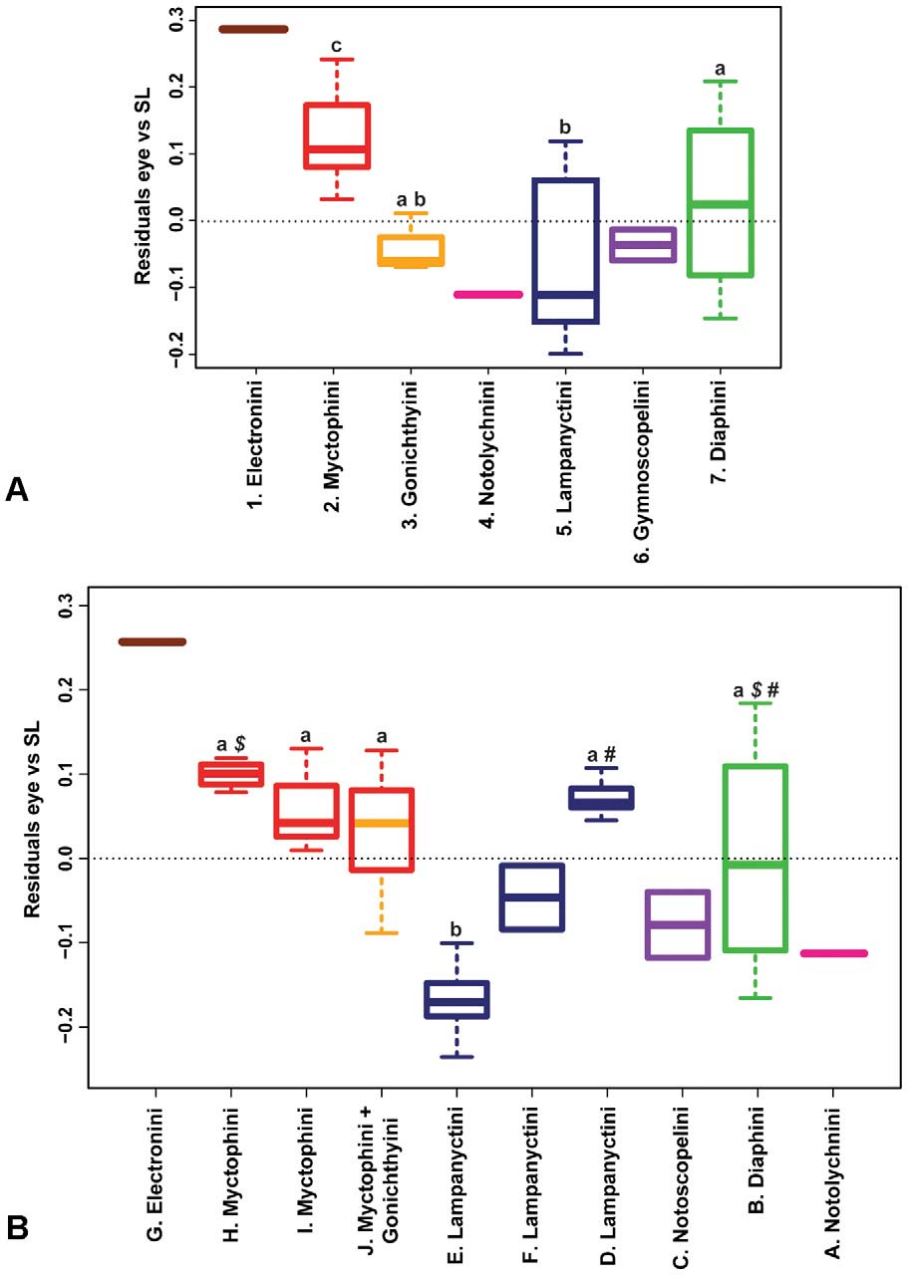
Residuals eye size corrected for body size of Myctophidae after correcting for phylogeny: (A) by tribes (phylogeny A, [43]) and (B) by clades (phylogeny B, [44]). Colours represent the tribes of Paxton et al. [43] for comparison, as in Fig. 4. Groups sharing the same superscript letter are not significantly different from one another based on the post-hoc analyses. Groups with no superscript letters were not included in the analysis due to the low samples size (n<3). #, *$* indicates genera that were significantly different for one of the ten randomly resolved polytomy phylogenies (*P_#_* = 0.04, *P_$_* = 0.03).

Phylogenetic ANOVAs reveal differences at both tribal (phylogeny A, n = 4, *F* = 12.4, *P* = 0.001, Figure 5A) and cladal (phylogeny B, n = 6, F = 14.33, *P* = 0.001; Figure 5B) levels. At the tribal level, post-hoc analyses revealed that the Myctophini possess significantly larger eyes than the other tribes analysed statistically and probably larger eyes than the tribes Notolychnini and Gymnoscopelini, which were excluded from the analysis due to insufficient sampling size (Figure 5A). Although also excluded from the statistical analysis for the same reasons, the tribe Electronini seems to possess the largest eyes. The tribes Diaphini and Lampanyctini are significantly different from each other, with the latter showing smaller eyes, but not significantly different from the Gonichthyini. At the cladal level (Figure 5B), most of the clades statistically analysed present similar eye sizes except clade E (Lampanyctini), which has significantly smaller eyes than all the other clades analysed. The three clades from the tribe Lampanyctini (D, E, F) do not overlap and show very different eye sizes, with clade D showing the largest eyes and clade E the smallest eyes. To the contrary, within the tribe Myctophini (H, I, J), all the clades possess similar eye sizes. As at the tribal level, Clade G (Electronini) did not possess sufficient observations to be included in the statistical analysis, but appears to be the clade showing the largest eye size.

**Figure 6:**
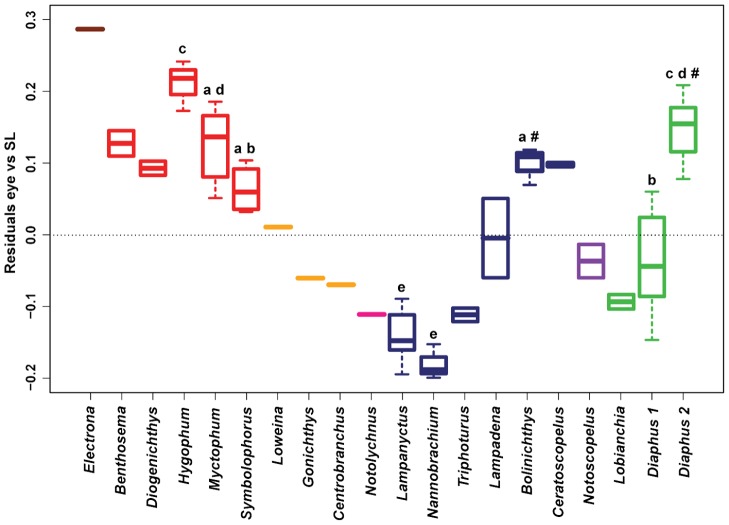
Residuals eye size corrected for body size by genera of Myctophidae, corrected for phylogeny (phylogeny A, [43]). Colors represent the tribes of Paxton et al. [43], as in Figure 4. The genus *Diaphus* was divided into two groups based on the presence/absence of the So. Groups with the same superscript letters are not significantly different from one another, independent of the phylogeny used based on the post-hoc analyses. Groups with no superscript letters were not included in the analyses due to the low samples size (n<3). # indicates genera that were not significantly different when analysed with phylogeny B (*P* = 1) and for four of the ten randomly resolved polytomy phylogenies of phylogeny A (*P* = 0.06–0.08).

**Figure 7:**
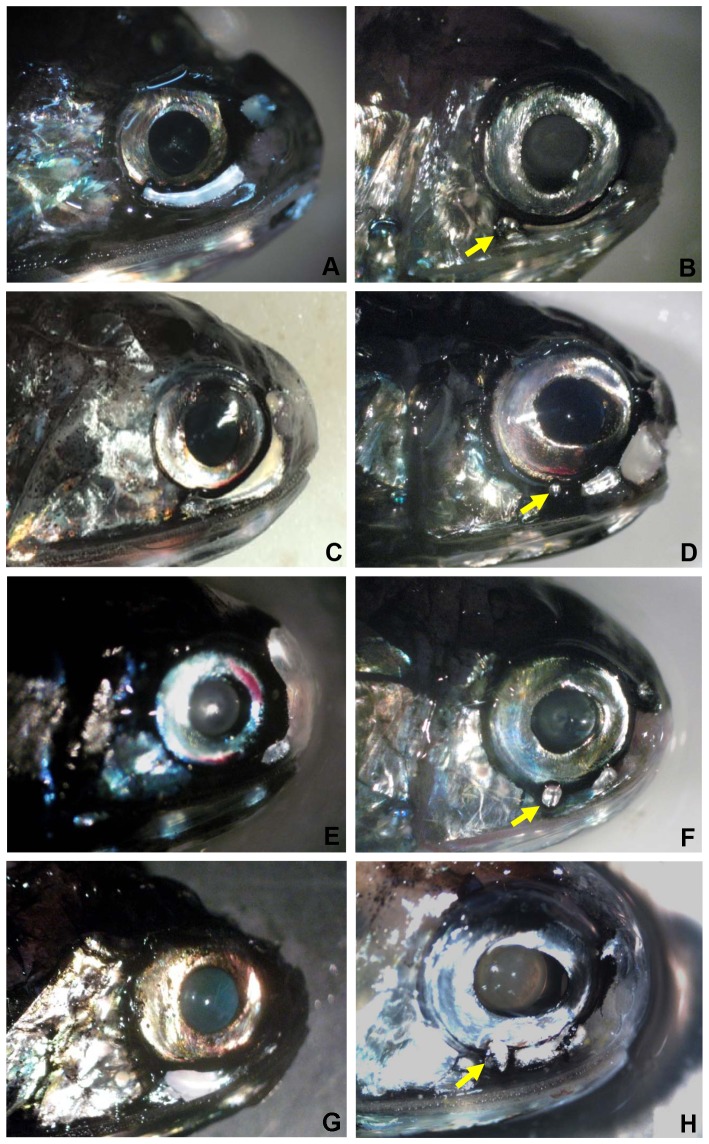
Variation in the size and location of the Dn, Vn and So luminous organs within the genus *Diaphus*. A = *D. luetkeni*, B = *D. brachycephalus*, C = *D. danae*, D = *D. mollis*, E = *D. phillipsi*, F = *D. parri*, G = *D. termophilus*, H = *D. holti*. A, C, E, G = *Diaphus* group 1 (So absent); B, D, F, H = *Diaphus* group 2 (So present). The yellow arrows indicate the position of the So photophore.

Eye-size variation is also observed between and within genera independently of the phylogeny used (n = 8, phylogeny A, *F* = 39.15, *P* = 0.001, Figure 6; phylogeny B, *F* = 41.54, *P* = 0.001). The results are similar between the two phylogenies except for two genera. When analysed with phylogeny A, *Diaphus* 2 is found to possess larger eyes than *Bolinichthys* (Post-hoc test, *P* = 0.028). However, no significant difference was found between the two groups when analysed with phylogeny B (Post-hoc test, *P* = 1). The genera *Lampanyctus* and *Nannobrachium* are not significantly different from each other, but possess significantly smaller eyes than the rest of the genera analysed. Despite belonging to different tribes/clades, several genera share similar eye sizes (i.e. *Myctophum*, *Bolinichthys*, *Diaphus* 2). Conversely, some genera belonging to the same tribe or clade show significantly different eye sizes (i.e. *Hygophum* and *Myctophum* from the tribe Myctophini and *Symbolophorus* and *Gonichthys* from clade I). Genera from the tribe Gonichthyini (*Loweina*, *Gonichthys*, *Centrobranchus*) appear to have smaller eyes than all the genera present in the tribes Electronini and Myctophini and similar eye sizes to the other tribes. A great variation in eye size is also observed within the same genus, i.e. *Diaphus*. This genus possesses great variability in Dn/Vn luminous organs (Figure 7) and can be divided further into two groups based on the presence or absence of one light organ, the So just below the eye. The two groups were found to be significantly different in term of eye size, with *Diaphus* species possessing an So (*Diaphus* 2) having larger eyes than *Diaphus* species without an So (*Diaphus* 1).

### Relationship between morphometric and ecological traits

Phylogenetically controlled multiple linear regression models do not reveal any significant relationships or trends between eye diameter, corrected for standard length, and any of the ecological variables examined in this study (Table 5). This lack of any significant relationships between eye diameter and ecological traits persists regardless of the phylogeny used in the analysis (Table 5).

**Table 5:** Regression model of eye diameter with different predictor variables when controlling for phylogeny (PGLS).

Phylogeny	n	*λ*	Predictor variables	*β*	*t-value*	*P*
Phylogeny A	58	0.939^*,*^	Standard length	0.925	11.927	**<0.001**
			Dn/Vn luminous organs	−0.012	−0.211	0.834
			Caudal luminous organs	−0.120	−1.485	0.144
			Luminous patches	0.017	0.343	0.733
			Luminous tissue sexual dimorphism	0.052	1.259	0.214
			Day depth	0.008	0.408	0.685
			Night depth	−0.013	−0.434	0.666
Phylogeny B	58	0.935^*,*^	Standard length	0.922	12.123	**<0.001**
			Dn/Vn luminous organs	−0.043	−0.717	0.477
			Caudal luminous organs	−0.197	−1.874	0.067
			Luminous patches	−0.025	−0.636	0.528
			Luminous tissue sexual dimorphism	0.047	1.178	0.244
			Day depth	0.008	0.419	0.677
			Night depth	−0.039	−1.451	0.153

The results are presented for one of the ten randomly selected polytomy resolved phylogenies for the two different phylogenies. Standard length was added as a covariate in the models. n = sample size, λ = phylogenetic scaling parameter, the superscript * after the parameter λ indicates whether the parameter was significantly different from 0 (first position) and from 1 (second position) in the likelihood tests, β = partial regression slope. The significance levels are shown in bold.

## Discussion

The aim of this study was to assess the variability in eye size within a range of species of lanternfishes from different depths to assess the influences of both ecology and phylogeny. To our knowledge, this is the first study to examine eye-size variability within the same family of vertebrates using representatives of more than 50% of the recognised genera.

### Eye-size variation and ecology

With respect to assessing whether the variation in eye size observed within the lanternfishes could be explained by ecological differences using phylogenetic comparative analyses, no significant relationship was found between relative eye size and any of our predictor variables. Our hypothesis that lanternfish species with a deeper distribution and/or less luminous tissues will have smaller eyes could not be validated with our dataset. This hypothesis was posed under the assumption than the gradual change in the visual scene in the mesopelagic zone will lead to a great diversity in eye size [5], [16], [4]. At depths where both bioluminescence and downwelling sunlight are present, as in the mesopelagic zone, adaptations of the eye toward the detection of one or the other will depend on the ecological task required. Adaptations to assess the intensity of downwelling light are essential to aid a species to hold a depth station during the day, to camouflage themselves by counter illumination, to vertically migrate, to set their circadian rhythm and/or to detect the presence of other animals above them. Adaptations for viewing bioluminescent signals will be an advantage in detecting other individuals (prey, predator, mate) in deeper zones, where bioluminescent cues predominate.

In addition to the ventral photophores, lanternfishes possess a number of luminous tissues that are thought to have several functions. Caudal luminous organs play a role in either escape responses by producing a blinding flash [56] or in sexual communication, where those organs are sexually dimorphic. Several hypotheses have been proposed for the function of the Dn/Vn organs in myctophids. They may be used as a head torch to search for prey [57], [56], to compare the intensity of their own photophore emissions with the downwelling sunlight [58] and/or for intraspecific communication in sexually dimorphic species. The function(s) of the other luminous patches remains unclear. However, the occurrence of sexual dimorphism in some of those patches suggests a role in sexual communication. Herring [33] concluded that the variety and complexity of sexual dimorphism in luminous organs were most likely due to their role in sexual signalling. The fact that some lanternfishes possess additional luminous patches and/or dimorphic luminous organs indicates than some species may rely more on bioluminescent signals than others. However, this hypothesis was not supported by our dataset in terms of eye size.

The visual capabilities of an eye are influenced by its size [59]. A larger eye will provide an advantage in the mesopelagic zone as it will increase the chance of photon capture, since a large eye normally has a greater pupillary aperture. However, the larger the eye the more energetically costly it will be [60]. Smaller eyes are less energetic and can act as a “distance filter” by reducing the visibility of a bioluminescent signal against a completely dark background (viewed within the bathypelagic zone and deeper, [61]). However, a small eye will be a disadvantage for species adapted for survival higher up in the water column, where high levels of background illumination are present and increased sensitivity is required. The results from this study indicate that lanternfishes show a great variability in eye size, independent of their depth distribution. While some species show the expected pattern by being strictly mesopelagic with relatively large eyes (i.e. *Myctophum nitidulum*), or venturing down into the upper parts of the bathypelagic zone during the day and possessing relatively small eyes (i.e. *Lampanyctus crocodilus*), others present an inverse pattern. For example, *Hygophum benoiti* possesses large eyes and frequents upper bathypelagic depths, while some *Lampanyctus* species with small eyes are restricted to the mesopelagic zone, with *L. omostigma* recorded at no deeper than 400 m (Supplementary Table S2, S3). These results suggest that some species may be clearly disadvantaged in term of vision by their small eye size in the mesopelagic zone (i.e. *L. omostigma*). After sampling very different eye types/sizes in the abyss, Murray & Hjort [62] wrote “Nothing has appeared more hopeless in biological oceanography than the attempt to explain the connection between the development of the eyes and the intensity of light at different depths in the ocean”. The conclusion of the present study seems to agree with this statement. However, the same analyses using light intensities rather than depth distribution might be more relevant here and will need to be considered in any future analyses, in addition to a more detailed analysis of the lanternfish visual system.

Even though the relative enlargement of the eye is an important adaptation for vision in dim-light conditions and for viewing bioluminescence, it is not the only visual adaptation found in the mesopelagic zone. Deep-sea fishes possess several other visual specialisations to enhance the sensitivity of the eye, especially at the level of the retina, that will need to be considered. Moreover, two types of light can be seen in the mesopelagic zone, bioluminescence and downwelling light, which do not require the same adaptations in terms of sensitivity for their detection. In fact, while sensitivity to bioluminescence (point-like light sources) is directly influenced by the size of the eye ([16], [10]), this is not the case for downwelling light (extended light source), where sensitivity is independent of eye size and is directly proportional to the size of the visual pixel (photoreceptor diameter, [63]). As a result, small-eyed species could potentially have a greater sensitivity to downwelling light than larger-eyed species depending of the diameter of their photoreceptors. This stresses the point that the visual capabilities of a species cannot be solely assessed by the size of the eye and that a number of other physical factors in addition to the type of visual stimulus might need to be taken into consideration in order to assess relationships with environmental variables. The question that then remains is: Do small-eyed lanternfishes really have a limited visual system or do they compensate for their small eye size with other visual specialisations? Very few studies have examined the visual system of myctophids. However, the group appears to have evolved eyes designed to enhance sensitivity, i.e. with an aphakic gap (*Tarletonbeania crenularis*, [58]), a pure-rod retina (*Lampanyctus crocodilus* [64]; *Lampanyctodes* sp [65]; *Stenobrachius leucopsarus* [66]), a tapetum lucidum (*Stenobrachius leucopsarus* [66]), a high photoreceptor density (*Lampanyctus crocodilus* [64], *Lampanyctodes* sp [65]; *Stenobrachius leucopsarus* [66]), visual pigments tuned to view bioluminescence (58 species, [67]), and a rather unspecialised retina with poor acuity (*Lampanyctus macdonaldi, Myctophum punctatum*, [68], [11]). However, these data have been compiled from very few species and most of the studies have only examined one or a few of these characteristics, which negated their inclusion in our analysis. It is also possible that small-eyed species have adapted to the mesopelagic zone in other ways by relying less on vision and more on other sensory systems. This hypothesis seems plausible considering the extremely high number of myctophid species present in the mesopelagic zone (∼250) and the quantitative differences in the size of the optic tecta [9].

### Eye-size variation and phylogeny

Results from this study showed great differences in eye sizes within the Myctophidae at all phylogenetic levels. At the subfamilial level both small and large eyes are present in the two different subfamilies, indicating that eye-size variations may have evolved several times within the family. Paxton [31] discussed the evolution of the Myctophidae and its two subfamilies and considered the Neoscopelidae the more generalised of the two families and the Myctophinae closer to the ancestral state than the Lampanyctinae, while admitting the larval characters indicated the Myctophinae were more specialised [45]. Poulsen et al. [44] presented the first molecular phylogeny for the family, but failed to resolve this question, with the exception of *Notolychnus,* which was used as the ancestral species of the two subfamilies. *Notolychnus valvidiae* is a small eyed species, suggesting that small eyes represent the ancestral condition and that larger eyes might have evolved several times within the lanternfish family.

The estimation of the phylogenetic signal by Pagel's lambda revealed that relative eye size within the family has a strong phylogenetic signal independent of the phylogeny used. By definition, members of a taxon are more closely related to one another than to any members of another taxon. Based on this definition, and if relative eye size is a relatively conserved variable over time, as Pagel's lambda seems to indicate, then eye size within a taxon (tribe or clade) would be more similar than between taxa and as a result, eye size of a species might be estimated simply based on the phylogenetic position of this species. In this sense, the sub-division of Paxton's tribe Lampanyctini into three different clades by Poulsen et al. [44] appears to be supported by our relative eye-size data, since clades D, E and F present very different eye-size ranges that do not overlap. However, great variation in eye size is also observed within the single genus *Diaphus* in our study. Kawaguchi and Shimizu [69] presented a taxonomic key for *Diaphus*, which divided the genus into four groups (SuO-group, So group, Ant-group and Dn-Vn group) based on the presence or absence of different luminous organs associated with the eye. The presence of the So below the eye is one of the first identifying characters used by several taxonomic keys to identify *Diaphus* species [32], (Paxton and Williams, unpublished data). Further division of this genus based on the absence or presence of the So in our study (*Diaphus* 1, *Diaphus* 2) shows that species possessing an So photophore have significantly larger eyes than species without this photophore, indicating a possible subdivision of this taxon based on the So photophore. Future molecular, morphological phylogenetic analyses will hopefully shed more light on this large complex group, which comprises some 75 species. The subdivision of the tribe Myctophini and inclusion of the tribe Gonichthyini within the Myctophini by Poulsen et al. [44] seems less obvious from our results. Relative eye sizes between Myctophini and Gonichthyini using Paxton et al.'s phylogeny are significantly different and do not overlap, indicating that members of the Gonichthyini have systematically smaller eyes than members of the Myctophini. We realise that eye size is only one character in the evolution of the group and our data set does not include all genera of either tribe.

### Limits of the study

#### Sampling methods

In this study, an attempt was made to categorize species according to their diurnal and nocturnal depth ranges. This task was complicated because of the lack of accurate depth-distribution data in the literature. Unfortunately, very few studies accurately estimate the depth at which a specimen is sampled, with the minority of sampling using opening-closing devices. Moreover, even fewer studies report depth distribution by life stages. Karnella [27] is to date the most comprehensive lanternfish depth-distribution study, where fishes were sampled seasonally from the Ocean Acre in the Northern Sargasso Sea at day and night using predominantly discrete-depth sampling gear every 50 m. Results from this study present accurate depth-distribution data by life stages for 20 of our 61 species. In addition to the lack of accurate depth-distribution data in the literature, several other issues make the task of depth categorisation of species challenging. In addition to the high level of interspecific variability in depth pattern, there is observed intraspecific variability in depth distribution depending on the season [27], moon cycle (i.e. *Hygophum hygomii* [70]), changes with size/age where larger/older specimens become non-migrant (i.e. *Benthosema glaciale* [27]) and ocean physics (i.e. *Lampanyctus crocodilus* [71]). Studies will often record the depth of a species at a specific location, season and time, which might not reveal the general pattern for the species. Finally, lanternfishes are able to efficiently avoid the net during the day [72], increasing the chances of biased data for those species. As a consequence, our depth categorisation may not represent the true distribution of several species, especially in cases where information in the literature was from a different ocean than the species analysed in our study.

### Phylogeny

Phylogenetic comparative analyses are dependent on the phylogeny used. To allow the most accurate analyses, a fully resolved phylogeny with branch lengths is usually required. Unfortunately, the phylogeny of the lanternfish family remains poorly resolved, even though they represent one of the most important groups of mesopelagic fishes in the ocean in term of biomass and energy transfer [73]. Nevertheless, the first molecular phylogeny for the family presented by Poulsen et al. [44] appears to support the basic morphological phylogeny of Paxton et al. [43], even though some differences in tribal arrangement are recognised. A better resolved phylogeny will undoubtedly improve our understanding of the relationship between the different tribes/clades and the variation in eye size, especially as more ecological data is accumulated (i.e. diet: luminescent prey vs non-luminescent) for this group.

### Conclusion

A great variability in relative eye size within the Myctophidae was observed at all taxonomic levels. However, variability in eye size within the family could not be explained by ecological variables (bioluminescence and depth patterns) in this study and seems instead to be driven by phylogenetic parameters. Further analyses including other environmental variables (i.e. diet, prey, light intensities) and a more complete phylogeny are needed to understand the great variability in eye size within myctophids. Moreover, examination of the visual system in more depth will be an essential step in assessing the visual capabilities of each species and to shed light on the visual adaptations of the lanternfish family in relation to their environment.

## Supporting Information

Table S1 **Analyses of covariance (ANCOVA) of the eye diameter versus standard length between juveniles and adults in Myctophidae.** n = sample size, β = partial regression slope. Only species with at least three observations for each stage (juvenile, adult) were analysed. Significant differences are shown in bold. The slopes are not significantly different between juveniles and adults.
(DOC)

Table S2 **Summary of the juvenile-adults night and day depth ranges found in the literature for the 61 species of Myctophidae studied.** The sampling area and depth range of our study samples is also given (N = night, D = day). Abbreviations for the areas can be found in Table S3. * Study using an opening-closing device.
(DOC)

Table S3 **List of abbreviations used for the areas sampled in Table S2.**
(DOC)
