# Supplementary material for: Eye-Size Variability in Deep-Sea Lanternfishes (Myctophidae): An Ecological and Phylogenetic Study
Source: PLoS One. 2013 Mar 5;8(3):e58519. doi: 10.1371/journal.pone.0058519 (PMC3589346; doi:10.1371/journal.pone.0058519)
Supplement: Table S1 — Analyses of covariance (ANCOVA) of the eye diameter versus standard length between juveniles and adults in Myctophidae. n = sample size, β = partial regression slope. Only species with at least three observations for each stage (juvenile, adult) were analysed. Significant differences are shown in bold. The slopes are not significantly different between juveniles and adults. (DOC) [file pone.0058519.s001.doc]

Table S1.

| **Species** | **n juveniles** | **n adults** | **Predictors** | ***β*** | ***T-value*** | ***P*** |
| --- | --- | --- | --- | --- | --- | --- |
| *Ceratoscopelus warmingii* | 3 | 8 | SL | 0.92 | 6.93 | **<0.001** |
|  |  |  | stage | -0.03 | -1.30 | 0.23 |
|  |  |  |  |  |  |  |
| *Lampanyctus alatus* | 5 | 4 | SL | 1.47 | 4.34 | **0.005** |
|  |  |  | stage | 0.05 | 1.00 | 0.35 |
|  |  |  |  |  |  |  |
| *Lampanyctus parvicauda* | 3 | 6 | SL | 0.91 | 9.53 | **<0.001** |
|  |  |  | stage | -0.05 | -1.21 | 0.27 |
