# Supplementary material for: Eye-Size Variability in Deep-Sea Lanternfishes (Myctophidae): An Ecological and Phylogenetic Study
Source: PLoS One. 2013 Mar 5;8(3):e58519. doi: 10.1371/journal.pone.0058519 (PMC3589346; doi:10.1371/journal.pone.0058519)
Supplement: Table S2 — Summary of the juvenile-adults night and day depth ranges found in the literature for the 61 species of Myctophidae studied. The sampling area and depth range of our study samples is also given (N = night, D = day). Abbreviations for the areas can be found in Table S3. * Study using an opening-closing device. (DOC) [file pone.0058519.s002.doc]

Table S2.

| **Species** | **Sampling depth** | **Sampling area** | **Night range** | **Day range** | **Area** | **References** |
| --- | --- | --- | --- | --- | --- | --- |
| *Benthosema glaciale* | 100-200 N | wMed | 750-1250 / 12-800 | 750-1050 / 375-800 | wNAtl / Med | [1]*/ [2] |
| *B. suborbitale* | 0-200 N | wAus | 0-100 | 600-700 | wNAtl | [1]* |
| *Bolinichthys longipes* | 87-107 N | CorS | 50-150 | 525-725 | cNPac | [3] |
| *B. nikolayi* | 202-429 N | CorS | <300 | <500 | cEPac | [4] |
| *B. supralateralis* | 500 N | CorS | 200-700 / 60-700 | 600-650 / 250-600 | wNAtl / GMex | [1]*/ [5]* |
| *Centrobranchus andreae* | 100 N | CorS | 100-165 | 640-650 | cNPac | [6] |
| *Ceratoscopelus maderensis* | 65 N | wMed | 33-1000 / 12/800 | 751-1000 / 100-1000 | wNAtl / Med | [1]*/ [2] |
| *C. warmingii* | 83-300 N | CorS | 0-500 | 800-1550 | wNAtl | [1]* |
| *Diaphus brachycephalus* | 202-329 N | CorS | 150-225 / 50-300 | 250-450 / 300-600 | wNAtl / GMex | [1] */ [5]* |
| *D. danae* | 253-377 N | CorS | 0-300 | 300-650 | Tas | [7]* |
| *D. fulgens* | 500 N | CorS | 85 | 1000 | ChiS | [8] |
| *D. garmani* | 100-150 N | CorS / wAus | surface / 0-125 | <225 / 325-750 | ePac / SAfr | [9] / [10] |
| *D. holti* | - | wMed | 80-235 | 500-675 | Med | [2] |
| *D. luetkeni* | 202-325 N | CorS | 400-800 / 60-300 | 500-800 / 300-600 | wNAtl / GMex | [1]* / [5]* |
| *D. meadi* | 50-100 N | wMed | 0 | 1250 | Tas | [11]* |
| *D. mollis* | 202-429 N | CorS / wAus | 0-700 / 33-350 | 100-800 / 300-500 | wNAtl | [1] */ [2] |
| *D. parri* | 200-329 N | CorS | 0 | 900 | Aus | [12] |
| *D. phillipsi* | 107 N | CorS | 50-200 | 600 | SAfr | [10] |
| *D. regani* | 50-400 N | CorS | 0 / <50 | 1000 | Aus | [12]/ [13] |
| *D. splendidus* | 107 N | CorS | 51-250 / 30-550 | 501-650 / 300-600 | wNAtl / GMex | [1] / [5] |
| *D. termophilus* | 200-477 N | CorS | 40-225 / 75-150 | 325-850 | eNAtl / GMex | [2] / [5]* |
| *D. whitleyi* | 329 N | CorS | 136-152 | 170-710 | Phi | [14] |
| *Diogenichthys atlanticus* | 50-150 N | wAus | 20-1000 / 50-700 | 500-1000 / 350-700 | wNAtl / GMex | [1]* / [5]* |
| *D. laternatus* | 36-146 N | PCT | Surface / >50 | 100-500 / 200-400 | eNPac / eSPac | [15]/ [16] |
| *Electrona risso* | 330-1000 D | wMed | 400-500 / 0-200 | 700-750 / 600-700 | Med / cAtl | [17] / [18]* |
| *Gonichthys tenuiculus* | Surface N | CPT | surface | - | cPac | [15] |
| *Hygophum benoiti* | 410 D | wMed | 18-1050 | 450-1100 | wNAtl | [1]* |
| *H. hygomii* | 0-202 N | wAus | 10-300 / 0-1000 | 400-800 / 500-1000 | eNAtl / wNAtl | [2]/ [1]* |
| *H. proximum* | Surface N | CorS / PCT | 25-150 | 500-700 | cNPac | [3] |
| *Lampadena luminosa* | 325 N | CorS | 75-250 / 65-600 | 525-725 / 500-1000 | cNPac / GMex | [3] / [5]* |
| *L. urophaos* | 200 N | CorS | 50-600 | 600-750 | wNAtl | [1]* |
| *Lampanyctus alatus* | 202-286 N | CorS / wAus | 50-300 | 700-850 | wNAtl | [1]* |
| *L. crocodilus* | 600 D | wMed | 50-950 / 1200 | 750-1000 / 1200 | wNAtl / Med | [1]*/ [19] |
| *L. iselinoides* | 450-707 D | PCT | <70 | - | ePac | [9] |
| *L. nobilis* | 107 N | CorS | 40-600 / 100-200 | 800-1000 / 300-<900 | GMex / wNAtl | [5] / [1] |
| *L. omostigma* | 36-146 N | PCT | <50 | 200-400 | eSPac | [16] |
| *L. parvicauda* | 61-302 N / 610-1200 D | PCT | 0-150 | - | GCal | [20]* |
| *L. pusillus* | 100-200 N | wMed | 50-325 / 50-1000 | 500-1000 / 550-850 | Med / wNAtl | [2] / [1]* |
| *L. vadulus* | 100 N | CorS | 0 | 1000 | Aus | [12] |
| *Lobianchia dolfleini* | 65 N | wMed | 25-400 | 375-600 | Med | [2] |
| *L. gemellari* | 107-329 N | CorS | 20-210 / 50-600 | 300-450 / 400-850 | GMex / wNAtl | [5]* / [1]* |
| *Loweina interrupta* | 100-150 N | wAus | 60-800 | - | wNAtl | [2] |
| *Myctophum asperum* | Surface N | CorS | Surface-150 / 0-125 | 400 / 425-750 | GMex / SAfr | [5]* / [10] |
| *M. aurolaternatum* | Surface N | CorS | 0 | 1000 | eAus | [12] |
| *M. brachygnathum* | Surface N | CorS | - | 280-340 | Phi | [14] |
| *M. lychnobium* | Surface N | CorS | 0 | 100 | eAus | [12] |
| *M. nitidulum* | Surface N | PCT | 0-15 / surface | 600-800 | cNPac / eNpac | [3] / [15] |
| *M. obtusirostre* | Surface N | CorS | Surface-150 / 0-15 | 500-600 / 500-700 | GMex / cNPac | [5]* / [3] |
| *M. spinosum* | Surface N | CorS | 0-15 | 600 | cNPac | [3] |
| *Nannobrachium cf. nigrum* | 200-300 N | CorS | 100-310 | 640-900 | cNPac | [3] |
| *N. idostigma* | <61 N | PCT | >50 | 250 | eSPac | [16] |
| *N. phyllisae* | <108 N | PCT | <300 | - | ePac | [9] |
| *Notolychnus valdiviae* | 211-348 N | CorS | 50-250 / 30-1050 | 350-1050 / 400-850 | GMex / wNAtl | [21]* / [1]* |
| *Notoscopelus elongatus* | 248 N, 414 D | wMed | 45-150 | 375-1000 | Med | [2] |
| *N. kroeyerii* | 366-500 D | eNAtl | 0-200 | 325->1000 | eNAtl | [2] |
| *Symbolophorus cf. boops* | Surface N | CorS | 0 | 900 | eAtl | [22] |
| *S. evermanni* | Surface N | CorS | 0-125 | 600-900 | cNPac | [3] |
| *S. rufinus* | Surface N | CorS | 0-125 / 0-900 | 425-850 / 750-900 | SAfr / wNAtl | [10] / [1]* |
| *S. veranyi* | Surface N | wMed | 0-150 | 100-700 | Med | [2] |
| *Triphoturus nigrescens* | 321 N | PCT | 200-1000 | 400-1000 | cNPac | [23] |
| *T. oculeus* | 5-290 N <450 D | CorS | >50 | >100 | eSPac | [16] |

References

1. Karnella C (1987) Family Myctophidae, lanternfishes. In: Gibbs RH, Krueger WH, editors. Biology of midwater fishes of the bermuda Ocean Acre. Washington, D.C.: Smithsonian Institution Press. pp. 51-168.

2. Hulley PA (1984) Myctophidae. In: Whitehead PJP, Bauchot M-L, Hureau J-C, Nielsen J, Tortonese E, editors. Fishes of the North-eastern Atlantic and the Mediterranean: Unesco. pp. 510.

3. Clarke TA (1973) Some aspects of the ecology of lanternfishes (Myctophidae) in the Pacific Ocean near Hawaii. Fishery Bulletin 71: 401-434.

4. Hulley PA, Duhamel G (2009) A review of the lanternfish genus *Bolinichthys* Paxton, 1972 (Myctophidae). Cybium 33: 259-304.

5. Gartner JV, Hopkins TL, Baird RC, Milliken DM (1987) The lanternfishes (Pisces: Myctophidae) of the eastern Gulf of Mexico. Fishery Bulletin 85: 81-98.

6. Backus RH, Craddock JE, Haedrich RL, Shores DL, Teal JM, et al. (1968) *Ceratoscopelus maderensis*: Peculiar sound-scattering layer identified with this myctophid fish. Science 160: 991-993.

7. Williams A, Koslow JA (1997) Species composition, biomass and vertical distribution of micronekton over the mid-slope region off southern Tasmania, Australia. Marine Biology 130: 259-276.

8. Yang J, Huang Z, Chen S, Lee C (1996) The deep-water pelagic fishes in the area from Nansha Islands to the northeast part of South China Sea. Beijing: Science Press. pp. 58-141.

9. Wisner RL (1976) The taxonomy and distribution of lanternfishes (family Myctophidae) of the eastern Pacific Ocean: Navy Ocean Research and Development Activity. NORDA Rep. 3. 229 p.

10. Hulley PA (1986) Order Myctophiformes. Myctophidae. In: Smith MM, Heemstra PC, editors. Smiths' Sea Fishes. Heidelberg: Springer-Verlag. pp. 282-322.

11. Flynn A, Kloser R (2012) Cross-basin heterogeneity in lanternfish (family Myctophidae) assemblages and isotopic niches (*δ*^13^C and *δ*^15^N) in the southern Tasman Sea abyssal basin. Deep Sea Research Part I 69: 113-127.

12. Flynn AJ (2012) Ecology and Zoogeography of Lanternfishes (family Myctophidae). PhD thesis.: The University of Queensland.

13. Hartmann AR, Clarke A (1975) The distribution of myctophid fishes across the central equatorial Pacific. Fishery Bulletin 73.

14. Bourret P (1985) Poissoins teleosteens: Gonostomatidae, Sternoptychidae, et Myctophidae (MUSORSTOM II). Memoires du Museum National d'Histoire Naturelle 133: 55-82.

15. Ahlstrom EH, Stevens E (1976) Report of neuston (surface) collections made on an extended CalCOFI cruise during May 1972. Calif Coop Oceanic Fish Invest Rep 18: 167-180.

16. Cornejo R, Koppelmann R (2006) Distribution patterns of mesopelagic fishes with special reference to *Vinciguerria lucetia* Garman 1899 (Phosichthyidae: Pisces) in the Humboldt Current Region off Peru. Marine Biology 149: 1519-1537.

17. Nafpaktitis BG, Backus RH, Craddock JE, Haedrich RL, Robison BH, et al. (1977) Family Myctophidae. In: Gibbs Jr RH, Berry FH, Böhlke JE, Cohen DM, Collette BB et al., editors. Fishes of the Western North Atlantic: Memoir, Sears Foundation for Marine Research. pp. 13-265.

18. Kinzer J, Schulz K (1985) Vertical distribution and feeding patterns of midwater fish in the central equatorial Atlantic. I. Myctophidae. Marine Biology 85: 313-322.

19. Stefanescu C, Cartes J (1992) Benthopelagic habits of adult specimens of *Lampanyctus crocodilus* (Risso, 1810) (Osteichthyes, Myctophidae) in the western Mediterranean deep slope. Scientia Marina 56: 69-74.

20. Robison BH (1972) Distribution of the midwater fishes of the Gulf of California. Copeia 1972: 448-461.

21. Ross SW, Quattrini AM, Roa-Varon AY, McClain JP (2010) Species composition and distributions of mesopelagic fishes over the slope of the north-central Gulf of Mexico. Deep-Sea Research Part II 57: 1926-1956.

22. Hulley PA (1992) Upper-slope distributions of oceanic lanternfishes (family: Myctophidae). Marine Biology 114: 365-383.

23. Hulley PA (1986) A taxonomic review of the lanternfish genus *Triphoturus* Fraser-Brunner, 1949 (Myctophidae, Osteichthyes). Annals of the South African Museum 97: 71-95.
