# Supplementary material for: Eye-Size Variability in Deep-Sea Lanternfishes (Myctophidae): An Ecological and Phylogenetic Study
Source: PLoS One. 2013 Mar 5;8(3):e58519. doi: 10.1371/journal.pone.0058519 (PMC3589346; doi:10.1371/journal.pone.0058519)
Supplement: Table S3 — List of abbreviations used for the areas sampled in Table S2. (DOC) [file pone.0058519.s003.doc]

Table S3.

| **Abbreviation** | **Translation** |
| --- | --- |
| CorS | Coral Sea |
| PCT | Peru-Chile Trench |
| Med | Mediterranean Sea |
| wMed | Western Mediterranean Sea |
| Aus | Australia |
| wAus | Western Australia |
| eAus | Eastern Australia |
| Tas | Tasman Sea |
| ChiS | China Sea |
| Pac | Pacific |
| cPac | Central pacific |
| ePac | Eastern Pacific |
| cNPac | Central North Pacific |
| eNPac | Eastern North Pacific |
| eSPac | Eastern South Pacific |
| eAtl | Eastern Atlantic |
| wNAtl | Eastern North Atlantic |
| eNAtl | Western North Atlantic |
| cNAtl | Central North Atlantic |
| Phi | Philippines |
| GMex | Gulf of Mexico |
| GCal | Gulf of California |
| SAfr | South Africa |
